# Learning Faces: Similar Comparator Faces Do Not Improve Performance

**DOI:** 10.1371/journal.pone.0116707

**Published:** 2015-01-15

**Authors:** Scott P. Jones, Dominic M. Dwyer, Michael B. Lewis

**Affiliations:** 1 Department of Psychology, Anglia Ruskin University, Cambridge, United Kingdom; 2 School of Psychology, Cardiff University, Cardiff, United Kingdom; 3 School of Psychology, University of New South Wales, Sydney, New South Wales, Australia; University of Cambridge, UNITED KINGDOM

## Abstract

Recent evidence indicates that comparison of two similar faces can aid subsequent discrimination between them. However, the fact that discrimination between two faces is facilitated by comparing them directly does not demonstrate that comparison produces a general improvement in the processing of faces. It remains an open question whether the opportunity to compare a “target” face to similar faces can facilitate the discrimination of the exposed target face from other nonexposed faces. In Experiment 1, selection of a target face from an array of novel foils was not facilitated by intermixed exposure to the target and comparators of the same sex. Experiment 2 also found no advantage for similar comparators (morphed towards the target) over unmorphed same sex comparators, or over repeated target exposure alone. But all repeated exposure conditions produced better performance than a single brief presentation of the target. Experiment 3 again demonstrated that repeated exposure produced equivalent learning in same sex and different sex comparator conditions, and also showed that increasing the number of same sex or different sex comparators failed to improve identification. In all three experiments, exposure to a target alongside similar comparators failed to support selection of the target from novel test stimuli to a greater degree than exposure alongside dissimilar comparators or repeated target exposure alone. The current results suggest that the facilitatory effects of comparison during exposure may be limited to improving discrimination between exposed stimuli, and thus our results do not support the idea that providing the opportunity for comparison is a practical means for improving face identification.

## Introduction

It is commonplace to carry some form of photographic identification in modern society. This form of identification is required in a variety of environments from workplace entry to immigration. Moreover, photographs are frequently used as a means of identifying wanted persons—something exemplified in the way that, during the 2003 invasion of Iraq, soldiers were issued a set of playing cards depicting the faces of the most wanted members of Saddam Hussein’s government. However, despite the reliance on photographic identification, the ability of people to accurately match between a photograph and the individual in question is typically poor [2]. For example, the field study by Kemp, Towell, and Pike [3] found that supermarket staff failed more than 50% of the time to notice that customers had presented photo identification which did not depict that individual. This occurred despite the staff knowing that they were under observation and taking part in a study. The difficulty that people have in matching between images of unfamiliar faces has also been well documented in laboratory research even under ideal viewing conditions [4] and with good quality images [5]. Indeed, even though there is some suggestion that feedback training can improve matching performance with unfamiliar faces, this improvement is restricted to individuals with initially poor face matching ability, and substantial error rates persisted even after training [6]. More generally, there is a wealth of experimental evidence from laboratory tests indicating that the processing of images of unfamiliar faces is substantially inferior to that of familiar individuals, especially when changes in image quality are involved. In stark contrast, our ability to recognise a familiar face from a photograph or picture is good even after a long time [7] and under a variety of different viewing conditions (for reviews see [8, 9]). The fallibility of human face recognition when presented with unfamiliar individuals highlights the limited value of using photographic identification in many circumstances.

Many studies on face perception have examined the observed differences in processing between familiar and unfamiliar faces. For example, internal features (*e.g.*, the eyes, nose and mouth) have more influence than the external features (*e.g.*, hair or face outline) in the recognition of familiar faces while the influence for feature types is reduced or reversed for unfamiliar faces [10, 11]. Further, it has been shown that it is easier to detect a difference between two pictures which are familiar compared to when they are novel [12], and that recognition is superior for familiar faces compared to unfamiliar faces using poor quality images [13]. Moreover, changes in viewpoint or expression for familiar faces have less impact than the same changes to novel faces [14].

Given the differences between familiar and unfamiliar face processing, one potential strategy to improve recognition of novel faces is to use a period of familiarisation training. For example, encouraging in-depth processing by making personality judgements can improve later recognition [15], as can giving relatively long periods of exposure to the to-be-remembered faces [16, 17]. In other cases, an improvement in face processing has also been reported using a comparatively small period of familiarisation. For example, Clutterbuck and Johnston [18] demonstrated that 10 two-second exposures produced better performance on a face matching task than was seen with novel images. This improvement was selective to the internal features of the face. In addition, relatively brief exposure (5 × 2 s) can facilitate discrimination between pairs of faces made similar by morphing them with each other [1, 19].

One explanation for these improvements is that they all offer the opportunity for comparison between stimuli. Indeed, Mundy *et al*. [1] demonstrated that it is the schedule by which stimuli were exposed, rather than simply the amount of exposure that determined the improvement in performance. For example, alternating or simultaneous exposure of different faces was more effective at facilitating discrimination between them compared to presentations of each face in separate blocks. Indeed, the facilitatory effects of intermixed exposure on subsequent discrimination are well attested to in the perceptual learning literature, and appear to be present across a wide range of stimuli and species (*e.g.*, visual stimuli in domestic chicks [20]; in humans with odours and flavours [21]; in rats with flavoured stimuli [22]). These schedule effects have been attributed to the fact that intermixed (or simultaneous) exposure supports a process of stimulus differentiation, whereby the effectiveness of stimulus-unique features that are useful in distinguishing the stimuli are enhanced, relative to features common to both stimuli [23, 24]. Although there have been numerous attempts to unpack the mechanisms by which stimulus differentiation might operate (*e.g.* [25–28]), they all recognise that exposure schedules that afford comparison between the to-be-discriminated stimuli during exposure will facilitate performance.

In all of the perceptual learning studies cited above, the key measure of performance was the ability to distinguish between particular stimuli that were presented during both exposure and test. Critically, in none of these cases was the ability to distinguish between an exposed stimulus and a novel foil compared to the ability to distinguish between two novel stimuli. Indeed, there is some evidence that implies comparison does not facilitate discrimination between exposed and nonexposed flavour stimuli in rats [29]. In the context of face processing and the playing cards depicting wanted members of Saddam Hussain’s Iraqi government, this means that playing with the Iraqi cards may only help to distinguish between two people on the wanted list and not between a person on the wanted list and a member of the general population, which was more likely to be the goal of issuing the cards.

Another issue with applying perceptual learning to face recognition is that unfamiliar face recognition is image-based [14, 30]. As such, it is sensitive to changes in lighting [31, 32], expression [14], and viewpoint [33] (for a review on factors affecting recognition see [9]). It is difficult to gauge, therefore, whether any previously demonstrated effects of comparison might reflect image-based learning or a more general improvement in recognition. Thus, before any potential forensic application of stimulus differentiation can be assessed it is important to ascertain whether comparison improves recognition of a stimulus *per se* or whether it only improves performance with the particular images that were presented during the comparison process. To relate this to learning from the Iraq playing cards example, it remains unclear whether a person would be able to learn from those cards to recognise a person in any view other than one that was similar to that shown on the card.

There is, however, some recent evidence that suggests that it is possible to generalise comparison-based learning to novel stimuli. Dwyer and Vladeanu [34] reported that matching performance, using artificially generated faces, can be facilitated by alternating a target face with similar comparators during exposure. In Experiment 1, participants either received 12 two-second exposures to a target face in alternation with four similar comparator faces or no pre-test target exposure at all. In Experiment 2, participants received brief exposure to one target in alternation with four similar comparator faces, a second target with four different sex comparator faces, a third target was exposed without comparators, and a final target received no pre-test exposures at all. Both experiments found that face matching accuracy was improved by the presence of similar comparators during pre-test exposure. The improvement in matching of faces was attributed to processes of stimulus differentiation as has been discussed in the literature on perceptual learning [34]. That is, Dwyer and Vladeanu [34] suggested that the similar comparators and the target face shared a set of common features, and that adaptation of those common characteristics by exposure to the similar comparators would have enhanced processing of the unique features of the target face, thus facilitating subsequent matching performance.

Although the findings of Dwyer and Vladeanu [34] certainly seem to support the idea that the enhancement produced by comparison might extend to novel test stimuli, there are two caveats which limit the interpretation and potential generality of their findings. The first relates to the stimulus set, namely computer-generated faces. These have many of the same properties as faces and are recognisable as people, but it remains obvious to the participants that they are not true representations of real people and may be lacking some of the features of real faces. The consequence of this is that any comparison-dependent effects may be limited to such artificial stimuli. Indeed, if we briefly consider the face-space metaphor whereby face representations are locations within a multidimensional psychological space [35], then the artificial stimuli used in Dwyer and Vladeanu [34] may have been qualitatively different to actual faces because they might inhabit points in face-space that are quite atypical. This may have made the stimuli more recognisable; certainly some models of face-space would predict this outcome (for a discussion, see [36]). Furthermore, empirical evidence suggests that the more distinctive a face is then the more easily recognised it is [37], because it provides unusual cues that may encourage a more in-depth processing strategy [38, 39]. The second caveat relates to the non-target comparators: Dwyer and Valdeanu [34] generated the non-target comparators used in the exposure phase, and the novel test foils used in the test phase, in exactly the same fashion—in particular, both similar comparators and test foils were created by morphing away from the target face. As such, all non-targets will have the same common features, and so the ability for comparison-facilitated exposure to generalise to novel test stimuli might be restricted to situations in which the same common features are present in all stimuli—a situation that is unlikely to be true for real faces.

To summarise, it is unclear whether the beneficial effects of comparison will be seen using real photographic stimuli and if this effect can genuinely transfer to novel test stimuli in a manner that makes it a useful applied practice. Thus, the main aim of the current experiments was to explore the conditions by which the effect of comparison can be applied to learning a previously unfamiliar individual and to explore the ability of any such learning to generalise to novel test stimuli. All experiments involved an exposure period whereby target individuals were familiarised before participants were required to identify the targets within line-ups of faces (henceforth known as ‘arrays’). Arrays always displayed one target alongside previously unseen foils. To ensure any transfer was not merely based on the recognition of particular images the faces displayed in the test arrays were always subject to expression and contrast changes.

## Experiment 1

The main purpose of Experiment 1 (see Table 1 for the full design) was to examine whether alternating a target face with four other same sex comparison faces during exposure affected participants’ ability to select that target face from a test array of novel foil faces. This corresponds to the same sex condition shown in Table 1. Control conditions comprised the target faces alternated with different sex comparison faces (different sex condition), the target faces presented in alternation with a fixation cross (no-comparator condition), and target faces presented for one extended period equalling the total exposure time for the other conditions (single-exposure condition).

**Table 1:** Design of Experiment 1.

**Condition**	**Training sequence**	**Test Arrays (Morph)**	**Test Array (Non-Morph)**
Single Exposure	A, fp	Select A from an array of A, A5, A6, A7, A8	Select AH from an array of AH, AH9, AH10, AH11, AH12
No-Comparator	B, fp, B, fp, B, fp, B, fp. (× 4)	Select B from an array of B, B5, B6, B7, B8	Select BH from an array of BH, BH9, BH10, BH11, BH12
Same sex	C, C1, C, C2, C, C3, C, C4. (× 4)	Select C from an array of C, C5, C6, C7, C8	Select CH from an array of CH, CH9, CH10, CH11, CH12
Different sex	D, X1, D, X2, D, X3, D, X4. (× 4)	Select D from an array of D, D5, D6, D7, D8	Select DH from an array of DH, DH9, DH10, DH11, DH12

*Note*: A-D indicate target faces. 1–4 refer to comparator faces (*e.g.*, C1-C4 in indicate same sex comparator faces to target C, while X1 to X4 illustrate different sex comparator faces to target D). Exposure was repeated four times as indicated by × 4. fp denotes fixation points which were represented by a cross on screen. 5–8 in the morph array indicate previously unseen faces used as foils (*e.g.*, A5 to A8 are morphed foils for target A). AH-DH represents target individuals with an expression change in the non-morph array. 9–12 represent unseen foils present in the non-morph array (*e.g.*, BH9 to BH12 are non-morphed foils for target B).

**Figure 1:**
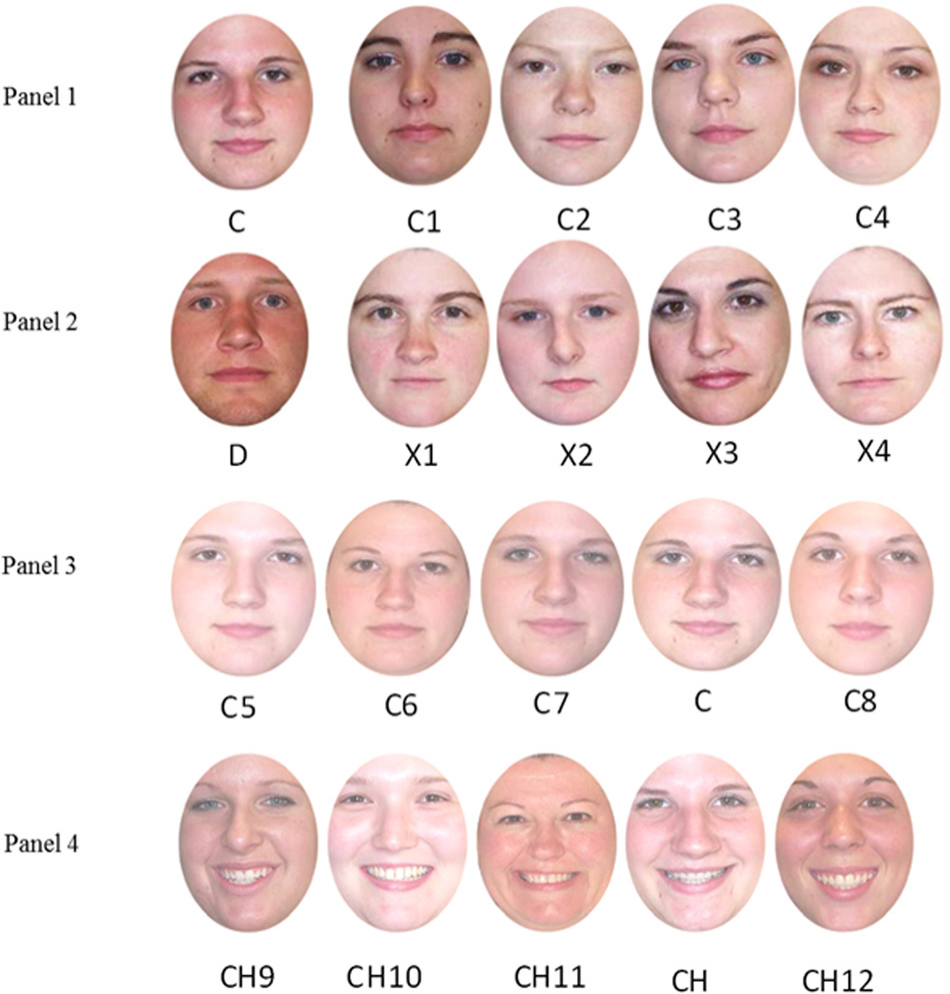
Examples of stimuli used in Experiments 1 and 2. Panel 1 represents a target face (*e.g.*, C) and same sex comparators (*e.g.*, C1–4) used in the exposure phase of Experiment 1. Panel 2 represents another target face (*e.g.*, D) and different sex comparators (*e.g.*, X1–4). Panel 3 displays an example of a morph array for target C (referred to in the Table 1 as C, and C5–8) used in the test phase. Panel 4 displays an example of the non-morph array for the target face C (referred to in the Table 1 as CH, CH5–8) used in the test phase. Participants were given a 3 s presentation of an array and then asked to choose the letter that corresponded to the target seen during exposure. For Experiment 2, same sex faces (*e.g.*, C1-C4) became dissimilar comparators and similar comparator faces were created using the same morphing technique as those in the morph array (*e.g.*, Panel 3).

We also examined whether the similarity between the target and the test foils influenced performance by manipulating whether the test foils were created by morphing from the target face to novel faces (see panel 3 of Fig. 1), or by using novel faces as foils (see panel 4 of Fig. 1). If comparison to similar faces does improve processing of a target face, as suggested by Dwyer and Vladeanu [34], then it is expected that targets alternating with same sex comparators will be better recognised than targets in other conditions.

### Methods

**Ethics Statement.** Participants in all studies reported here provided written informed consent. The research was approved by the Research Ethics Committee at Cardiff University (Title: Comparison in face processing. Ethics Code: EC.09.11.03.2294).

**Participants.** A total of 32 participants (27 females and 5 males, mean age 20.2, range 18–34), were recruited from the School of Psychology at Cardiff University. Participants received course credit in return for their participation. All had normal or corrected-to-normal vision.

**Apparatus and stimuli.** Eight white faces, four male and four female, were selected from the Centre for Vital Longevity Face Database [40] to be targets. Faces were between the ages of 19 and 45 years old and were selected so as to avoid the presence of non-face cues (*e.g.*, glasses and facial hair). Two images of each target face were selected. One image displayed a neutral expression that was used in the exposure phase and morphed test array. The other image displayed a happy expression used in the non-morphed test array only. Each target was grouped with twelve faces of the same sex. Four became same sex comparators and eight were used as test array foils. Four of the test array foils displayed a neutral expression (and were used in the morph test array) the other four displayed a happy expression (and were used in the non-morph test array). Different sex comparators were the opposite sex to the target face (*e.g.*, Panel 2 of Fig. 1). External features of all faces were removed by applying a mask function in Adobe Photoshop image editing software. Images were displayed centrally on a white background at 360 × 504 pixels subtending to an approximate visual angle of 12.5° × 17.2°.

All test arrays were constructed from faces of the same sex as the target. Two types of test array were used—both displaying the internal features of five individual faces. The first type was a morph array, in which the stimuli consisted of the target plus four morphed faces (Panel 3 of Fig. 1). The second type was a non-morph array, in which the target face and four other non-morphed faces all displaying happy expressions were presented (Panel 4 of Fig. 1). None of the foils in either array had been seen in any exposure condition. Faces in the morph array were created using a software package called Morpheus v3.10 professional, by morphing four previously unseen individuals towards the target face. Morphs were 50% blends of the target face and a non-target face. Both types of array were displayed on-screen at 1024 × 367 pixels, subtending an approximate visual angle of 32.4° × 12.8° with a screen resolution of 1024 × 768. All arrays were subject to a tonal change using the brightness/contrast adjustment tool in Adobe Photoshop image editing software. Each array was adjusted to +50% brightness and-20% contrast of the original image. This was applied to reduce the likelihood of participants being able to picture match from exposure to test. The stimuli were presented centrally on a 17 inch monitor. A custom-written programme using DirectRT software was used to control the presentation of the stimuli on a PC. Responses were registered using a computer keyboard with QWERTY layout. For the experiment, the letters A, S, D, F and G were covered with coloured labels A, B, C, D and E to match the letter depicting each face in the test array.

**Design and procedure.** Participants completed all four conditions (single, no-comparator, same sex, and different sex: see Table 1) in a within-subject design. Single exposure gave one presentation of a target face which remained on screen for thirty-two seconds (this was time-matched to the total amount of exposure time in the repeated exposure conditions). The repeated conditions (no comparator, same sex and different sex conditions) presented each target a total of sixteen times for two seconds each. For the same sex and different sex conditions, each target had four comparator faces. Each comparator was presented four times per condition, equating to a total of sixteen comparator presentations. Following exposure, participants were asked to select the target face from within both a morphed and a non-morphed array. This task was accompanied by a measure asking participants how confident they felt about their selection. There were two repetitions of each condition (one with a male and the other with a female target face) before moving on to the next condition.

Participants were sat approximately 70 cm from the computer screen. A brief practice trial gave an opportunity for participants to be familiarised with the general procedure of exposure/test phases. During the exposure phase, a randomly assigned name such as ‘Matthew’ was presented under each target face, while comparators for this target were labelled ‘Not Matthew’. Names were used to facilitate instructions to participants who were asked to identify named individuals during the test phase (*e.g.*, “*Please select the target face ‘Matthew’ from the following array*”). Participants were then given an array of faces which were displayed for 3 s. Following this array exposure participants were asked to make their identification. Confidence was then measured using a button response to a 7-point Likert scale (1: ‘Not at all confident’, 7: ‘Extremely confident’).

The assignment of faces to condition was counterbalanced so that all faces were presented an equal number of times within each condition. Furthermore, the presentation of arrays was counterbalanced such that half the participants received test trials with the morph array first. The other half of the participants saw the non-morph array first. Thus any carry-over effects between morph and non-morph trials cannot systematically influence the difference between these trial types. Moreover, the order of exposure conditions was also counterbalanced such that each condition was presented equally often first, second, third or fourth across participants.

**Data, Bayes, & Power Analysis.** The primary measure reported in all the experiments here is accuracy. A Confidence-Accuracy (CA) measure was also calculated by multiplying accuracy (negatively scored for incorrect answers so 1 = Correct and -1 = Incorrect) by the confidence score (less 0.5). This gives a score between -6.5 and +6.5 in 13 equal steps. This CA score highlights the fact that a highly confident incorrect answer demonstrates worse performance than low confidence incorrect answers while highly confident correct answers represent the best performance. While this CA measure could potentially have been more sensitive due to its greater range of possible scores, in all of the experiments reported here, the pattern of results observed with the CA scores was the same as with accuracy alone—demonstrating that the observed null effects for accuracy are not simply the results of a restricted range of possible scores. Because the CA and accuracy scores produce the same pattern of results, the CA measure will not be reported further. All data for each experiment reported are available as a supplementary file called S1 Data JDL.

In all experiments reported here, the absence of differences between different exposure conditions is potentially as informative as the presence of differences between conditions. It has always been the case that standard null-hypothesis significance testing only assesses how unlikely the observed data is given the assumption of the null hypothesis. As such, it does not provide a direct assessment of whether the absence of a significant difference can be taken as positive evidence for there being no true difference between conditions. One traditional way to address the problem is to use a power analysis—with the logic being that if an experiment which was appropriately powered relative to the predicted effect size failed to find a significant effect, then that would be strongly suggestive that there was no effect of the predicted size there to find. For the current experiments, the most important potential effect lies in the difference (or otherwise) between conditions where the target was exposed alongside similar/same sex comparators, and conditions where the target was exposed without such comparators. The most relevant prior result in this context is from perceptual learning studies examining comparison (*e.g.* [1]). In order to assess the ability of our experiments to detect a difference between similar/same sex conditions and control a power analysis was conducted, using G^*^Power software [43]. The relevant effect size for perceptual learning and comparison, in particular the size of the difference between intermixed (comparison) and blocked (non-comparison exposed control) conditions [1] was *f* = .53: a large effect by Cohen’s classifications [44]. Assuming this large effect size, and setting *α* at.05, the power (1—*β*) of our experiments to detect a comparison effect is as follows: Experiment 1 & 2 = .83, Experiment 3A = .90. In short, the current studies are well-powered to detect the sort of large effect sizes that would be both pragmatically important and expected on the basis of prior examination of comparison effects in perceptual learning. Given this, a failure to find a significant difference between the critical exposure conditions would suggest that the predicted large effect of this manipulation was in fact absent.

Although power analyses go some way to addressing how informative a null result might be, they make only limited use of the data actually obtained in the experiment (beyond asking whether it produced a significant difference or not). In contrast, Bayesian tests are based on calculating the relative probability of the null and alternative hypotheses, and thus afford the assessment of whether the evidence is in favour of either of these hypotheses. The Bayes factor (denoted as *B_01_*) relates to the ratio of probability for the observed data under a model based on the null hypothesis compared to a model based on some specified alternative model. The analyses we report here were performed using the web-based calculator (http://pcl.missouri.edu/) and utilised the Jeffreys-Zellner-Siow (JZS) prior suggested by Rouder *et al*. [41]. The JZS prior assumes a distribution of possible alternative effect sizes centred on zero where the bulk of possible effects are close to that centre. Although the effect sizes for comparison manipulations in perceptual learning studies are quite large (see power analysis discussion above), assuming large effect sizes for the alternative hypothesis would result in a very liberal Bayes factor estimate for the strength of evidence in favour of the absence of meaningful effects of comparison. Thus, using the default assumptions provides a relatively conservative test of whether the data favours the null hypothesis in the present case. The resulting Bayes factors can then be interpreted as either supporting the null or alternative (or as inconclusive evidence for either) according to the convention suggested by Jeffreys [42] and recommended by Rouder *et al*. [41]: a Bayes factor over 3 suggests there is some evidence to support the null hypothesis, while a factor of 10 indicates strong evidence for the null. On the other hand, a factor less than 1/3 suggests some evidence for the alternative and less than 1/10 indicates strong evidence favouring the alternative. Any value between 1/3 and 3 constitutes inconclusive evidence in support of either the null or alternative.

### Results and Discussion

**Figure 2:**
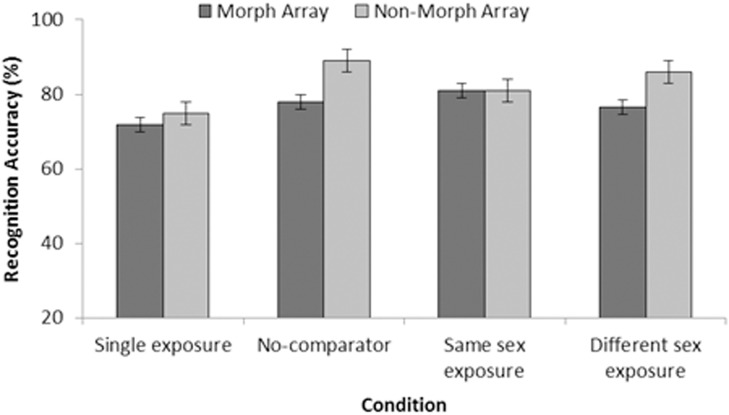
Test accuracy as percentage correct (with SEM) from Experiment 1. Data are organised by exposure condition (single, no-comparator, same sex, and different sex), and are presented as a function of array type (morph or non-morph).

Fig. 2 shows the test data as the percentage of correct target selections as a function of exposure condition (single exposure, no comparator, same sex exposure and different sex exposure), and test type (morph and non-morph). It is noticeable that there was little, if any, improvement in identification when target stimuli were exposed with same sex comparators. Thus, learning appeared to be unaffected by the opportunity for comparison between targets and same sex faces. A within-subject ANOVA confirmed this observation indicating no significant effect of exposure condition on accuracy *F*(3, 93) = 1.53, *p* = .212, MSE = 0.082. There was a significant main effect due to test *F*(1, 31) = 4.54, *p* = .041, MSE = 0.048, with the morph array producing less accurate responses than the non-morph array. No significant interaction was observed between type of exposure condition and type of test, *F*(3, 93) = 0.75, *p* = .524, MSE = 0.057. Despite the lack of a significant main effect of exposure condition, pairwise comparisons were computed in order to explore the performance of the individual exposure conditions and to facilitate Bayesian analysis. Turning first to the same sex comparator condition, which might have been expected to produce the best performance, there were no significant differences compared to: single exposure, *F*(1, 31) = 1.77, *p* = .194, MSE = 0.221, *B_01_ = * 3.157, no comparator, *F*(1, 31) = 0.25, *p* = .620, MSE = 0.140, *B_01_ = * 6.365, or different sex comparators, *F*(1, 31) = .00, *p* = 1.000, MSE < 0.01, *B_01_* = 7.299. There was also no difference between single exposure and the other conditions (no comparator, *F*(1, 31) = 3.43, *p* = .074, MSE = 0.192, *B_01_* = 1.493 and different sex, *F*(1, 31) = 2.26, *p* = .143, MSE = 0.173, *B_01_* = 2.519). Nor was there a difference between no comparator and different sex comparators (*F*(1, 31) = 0.28, *p* = .598, MSE = 0.124, *B_01_* = 6.358).

The accuracy test data is from a small number of trials for each participant in each condition. Although ANOVA methods are robust with respect to violations of its underlying assumptions, in order to assess whether the restriction in range of possible scores had a meaningful effect on the ANOVA reported above, the data was re-analysed using Friedman’s non-parametric methods for assessing differences between multiple within-subject conditions, and follow-up analyses were performed using Wilcoxon matched-pairs tests. There were no significant differences in recognition accuracy depending on exposure condition, *χ^2^*(3) = 3.19, *p* = .362. Post-hoc analysis revealed there were no significant differences in recognition between exposure conditions (largest *Z* = 1.67, *p* = .095 between no-comparator and single exposure). As these non-parametric analyses matched that of the ANOVA it would appear that the analysis reported in the main text has not been unduly influenced by the restriction of range in a way that affects the theoretical interpretation of the results. This issue will not be rehearsed at length in subsequent experiments, but the non-parametric analysis will be reported to confirm that the general argument made here is applicable to all experiments.

In short, the selection from an array of novel foils of target faces that had been exposed alongside same sex comparators was no more accurate than in any of the control conditions. Critically, Bayes analysis suggested genuine evidence in favour of the null in all of these cases. This result stands in apparent contrast with previous studies using artificially generated faces [34] or with studies of perceptual learning with morphed faces [1].

## Experiment 2

While Experiment 1 did not reflect previous evidence suggesting that comparison aids performance, numerous differences between the details of the experiments could have contributed to the observed results. Perhaps the most theoretically interesting difference between the experiments was the nature of the images used as comparators. The comparators used by Dwyer and Vladeanu [34] in their similar condition were created by morphing away from the target face. Thus, there was a strong degree of similarity between the target and similar comparators. Moreover, as the test foils were created in the same fashion then both similar comparators and test foils were constrained to have some of the same features in common (and in common with the target face). This level of similarity may have enhanced any process of differentiation between the target and foils. In Experiment 1, the conditions were based on sex and so even the supposedly “similar” (same sex) comparator faces may have been too far from the target face to support a useful comparison process. Indeed, the stimuli used by Mundy *et al*. [1] were also morphed faces that possessed a high degree of similarity. In addition, the original demonstration by Dwyer and Vladeanu [34] included a control whereby the target was presented for a brief period to provide a baseline for the accuracy of identification in the absence of repeated exposure prior to the matching test.

The design of Experiment 2 addressed these issues in a within-subjects design while otherwise retaining the same general procedural details and photographs as the previous experiment. Brief exposure gave a single (2 s) presentation of a target while the no-comparator condition remained unchanged from Experiment 1. Similar comparators were created by morphing away from the target (as with the test foils in the morph array from Experiment 1: see Panel 3 Fig. 1) while dissimilar comparators were the same sex as the target but with no other treatment (as with the same sex condition from Experiment 1, faces C1-C4 in Panel 1 Fig. 1). Following exposure, participants were required to identify the target from two separate test arrays (one with morphed foils, the other with unmorphed foils), and give confidence ratings for their identity choices. If the results of Experiment 1 are reliable then there should be no differences between the three repeated exposure conditions at test. Moreover, if repeated brief exposure facilitates processing as previous research has shown [18], then all repeated exposure conditions should produce more accurate selection of the target face from the test arrays than would brief exposure.

### Methods

**Participants**. A total of 32 participants, 28 females and 4 males (mean age 20.9, range 18–32), were recruited from the School of Psychology at Cardiff University. No participant had taken part in the previous experiment. All had normal or corrected-to-normal vision.

**Stimuli**. The target stimuli consisted of the same set used in Experiment 1. From this set targets were then separately morphed towards each comparator face. Similar comparators were 50% morphs of the target and a distractor face. Dissimilar comparators were unmorphed faces of the same sex. Thus, similar comparators were defined as 50% between a target and a comparator; dissimilar comparators were defined as the same sex as the target (*i.e.*, as for the same sex comparators from Experiment 1). As in Experiment 1, morphed faces had the external features removed by applying a mask function in Adobe Photoshop image editing software. Each photograph was displayed centrally on a white background at 360 × 504 pixels subtending to an approximate visual angle of 12.5° × 17.2°. All other details, such as the creation of the test arrays, were as in Experiment 1. It should be noted that although morph images were displayed in training and test, the comparison images were not the same identify as those used at test.

**Design and Procedure**. Participants completed all four conditions in a within-subject design: brief exposure, no-comparator, similar, and dissimilar exposure. Brief exposure consisted of a single 2 s exposure to a ‘target’ followed by the selection task. The design of the repeated conditions (no comparator, similar and dissimilar) was based on those of Experiment 1 other than the differences in stimulus definition. That is, each target was presented a total of sixteen times and both similar and dissimilar conditions had four comparator faces, with each comparator presented four times per condition, equating to a total of sixteen comparator presentations. All other procedural details remained consistent with Experiment 1, including the counterbalancing of the assignment of faces to condition, the order in which the exposure conditions were presented, and the order of testing in the morph and non-morph conditions.

### Results

**Figure 3:**
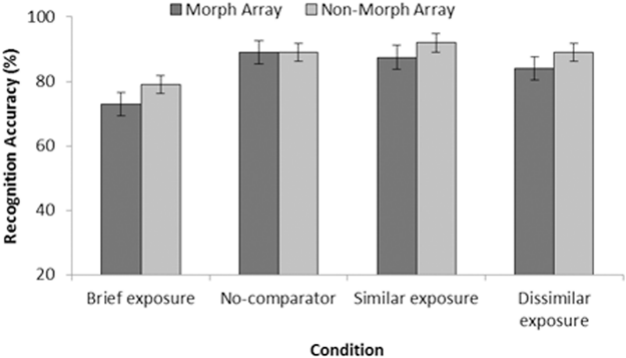
Test accuracy as percentage correct (with SEM) from Experiment 2. Data are organised by exposure condition (brief, no-comparator, similar, and dissimilar), and are presented as a function of array type (morph or non-morph).

Fig. 3 displays percentage of correct responses as a function of exposure condition (brief, no-comparator, similar and dissimilar) and test type (morph and non-morph). Performance was generally better following repeated exposure compared to brief exposure on both test arrays, but there is little or no difference between the repeated conditions on either array type. A within-subject ANOVA with factors of exposure condition (brief exposure, no comparator, similar exposure and dissimilar exposure) and test type (morph and non-morph) indicated a significant main effect on accuracy depending on exposure condition *F*(3, 93) = 3.60, *p* = .016, MSE = 0.067, but no other main effects or interactions (largest *F*(1, 31) = 1.90, *p* = .177, MSE = 0.051 for the main effect of test type). Pairwise analysis suggested that brief exposure produced lower accuracy than all other conditions: Similar exposure, *F*(1, 31) = 6.67, *p* = .015, MSE = 0.169 *B_01_* = 0.383, dissimilar exposure, *F*(1, 31) = 5.16, *p* = .030, MSE = 0.128, *B_01_* = 0.711 and the no comparator condition, *F*(1, 31) = 10.33, *p* = .003, MSE = 0.097 *B_01_* = 0.094. No differences were observed between other exposure conditions. That is, comparing the similar exposure to no comparator, *F*(1, 31) = 0.03, *p* = .876, MSE = 0.157, *B_01_* = 7.210, and dissimilar comparator, *F*(1, 31) = 0.72, *p* = .402, MSE = 0.087, *B_01_* = 5.155 found no advantage for similar comparators. Furthermore, there were no differences between the no-comparator and dissimilar comparator conditions, *F*(1, 31) = 0.21, *p* = .647, MSE = 0.164, *B_01_* = 6.576.

Non-parametric analysis these results confirmed the conclusions of ANOVA analysis. That is, there was a significant difference in accuracy between exposure conditions *χ^2^*(3) = 11.43, *p* = .010. Post-hoc analysis revealed that the brief exposure condition was recognised less accurately than the no-comparator *Z* = 2.82, *p* = .005, similar *Z* = 2.38, *p* = .017, and dissimilar exposure conditions *Z* = 2.29, *p* = .022. There were no differences between the other exposure conditions (largest *Z* = 0.85, *p* = .396 between similar and dissimilar exposures). As in Experiment 1, this analysis closely reflects that of the parametric analysis reported above.

### Discussion

As in Experiment 1, exposing the target along with similar comparators failed to produce superior selection of a target face from the novel test arrays as compared to exposure without similar comparators. Brief exposure to a target produced lower levels of accuracy than all other conditions. Thus, the absence of facilitation in the similar comparator condition cannot be attributed to a failure of learning *per se*. Similar patterns of performance have been observed in other learning tasks utilising a single vs. multiple exposure design (*e.g.*, Experiment 1 of Longmore, Liu, and Young’s research [30]). The absence of a comparator-similarity effect was observed despite the fact that a morphing procedure was used to ensure that the comparators were genuinely similar to the targets. These conclusions were supported by the Bayesian analysis which again provided evidence for the absence of an effect. Moreover, both the comparator faces and test foils were based on the target, ensuring that the general level of similarity between the target and both foils and comparators was the same. These methods closely replicate those used by Dwyer and Vladeanu [34] and thus the current results imply that insufficient comparator similarity alone cannot explain the absence of facilitation by similar comparators with real face images.

That said, the artificially generated faces used by Dwyer and Vladeanu may well come from a more restricted set of dimensions than the real face images used in the current experiments. Indeed, as face space has been estimated to contain between 15 and 22 dimensions [36], simply training a target against four comparators may not have spanned enough of this space to ensure that any particular dimensions which differentiated the target and comparators were the same dimensions which differentiated the target and novel test foils. Therefore, Experiment 3 examined whether the number, rather than simply the type, of potential comparators influenced learning of a novel face.

## Experiment 3A

In Experiment 3, all participants were exposed to same sex and different sex comparator conditions. Within each of these, target stimuli were shown in alternation with 0, 1, 2, 4, or 16 different comparators (3A) or briefly with one comparator (3B). As noted above, exposure to multiple different comparators should maximise the possibility that the dimensions (or features) on which the target differs from the comparators overlap with the dimensions on which the target differs from the test foils (Table 2 summarises the design of Experiment 3A).

**Table 2:** Design of Experiment 3.

**Condition**	**Target**	**Number of Comparators**	**Test Array**
Same sex	A	0	Select AH from a range of AH, AH1, AH2, AH3
	B	1	Select BH from a range of BH, BH1, BH2, BH3
	C	2	Select CH from a range of CH, CH1, CH2, CH3
	D	4	Select DH from a range of DH, DH1, DH2, DH3
	E	16	Select EH from a range of EH, EH1, EH2, EH3
Different sex	F	0	Select FH from a range of FH, FH1, FH2, FH3
	G	1	Select GH from a range of GH, GH1, GH2, GH3
	H	2	Select HH from a range of HH, HH1, HH2, HH3
	I	4	Select IH from a range of IH, IH1, IH2, IH3
	J	16	Select JH from a range of JH, JH1, JH2, JH3

*Note*: 0–16 represents the number of comparator faces displayed in alteration with the Target faces A—J. AH-JH represents target individuals with an expression change. 1–3 in the test arrays represents the different faces used as present in each array (*e.g.*, AH1 to AH3 are the test foils for target A). Note, this design was performed twice, once each with male and female target faces.

### Methods

**Participants**. A total of 40 participants, 33 females and 7 males (mean age 20.7, range 18–25), were recruited from the School of Psychology at Cardiff University, none of whom had previously completed the first two experiments. All had normal or corrected-to-normal vision.

**Stimuli**. A total of twenty front-view photographs, 10 male and 10 female, were selected from those freely available in the public domain to become target faces. For each target, a further 19 other faces were selected that were of the same sex and had similar hair colour and style. From the set, 16 became same sex comparators and 3 were used as foils. All individuals were white and aged between 18–25 years old. Half were male, and the other half female. Each image was cropped, resized, and converted to an 8-bit quality so that images had a standard width and height of 400 × 600 pixels, subtending to an approximate visual angle of 13.9° × 20.3°, during exposure. Each exposure stimulus displayed a neutral expression. The test arrays displayed a different image of the same target alongside three novel foils. Each array was displayed at 764 × 282 pixels subtending to a visual angle of 25.2° × 9.8°. All arrays were subjected to a contrast change as described in Experiment 1. All images retained some background information and external face features.

**Design and procedure**. Participants completed all conditions in a within-subject design. There were two conditions of exposure: Same sex exposure, in which a target alternated with same sex comparators, and different sex exposure, in which a target alternated with different sex comparators. Comparators were defined in the same way as Experiment 1: same sex and different sex in relation to the relevant target. Within each comparison condition there were five target faces which differed in the number of comparison faces that were presented with them (0, 1, 2, 4, or 16). In every case, the target was presented 16 times, and the comparison stimuli were interleaved between these presentations, with repetition of the comparators in the 1, 2, and 4 comparator conditions (see Table 2). All conditions were presented with both male and female target faces.

At the start of the experiment, participants were seated approximately 70 cm from the screen and instructed to examine the faces carefully and try to remember the target face presented. The presentation began with a set of standardised instructions shown on screen explaining the study. The presentation format and timings of stimuli were identical to those of Experiment 1 and 2. That is, exposure for each face was consistent with Experiments 1 and 2 (*i.e.*, 2 s with a 1 s ISI) and was followed by the selection task and finally a measure of choice confidence. For this experiment there was only one test array type (unmorphed faces). Responses were made in the same way as previous experiments, with the exception that response time was unlimited and a response could be made during the array presentation. Arrays disappeared when a response was made. The experiment was run in four blocks. Each block comprised one comparator condition (*i.e.*, same sex or different sex) and sex of the targets (*i.e.*, male or female). A block consisted of exposure to five target faces and the appropriate comparators. Each of the five targets in a condition was exposed with a different number of comparators (0, 1, 2, 4, and 16). Between blocks participants were able to pause before a new block was initiated by the experimenter. Within each block the order in which the five different comparator number conditions were presented was counterbalanced according to a Latin square procedure. In addition, across all participants, half were given the same sex conditions first and the other half were given the different sex condition first. The blocks then alternated between same sex and different sex comparators. Within this, male and female targets were presented first equally often.

### Results

**Figure 4:**
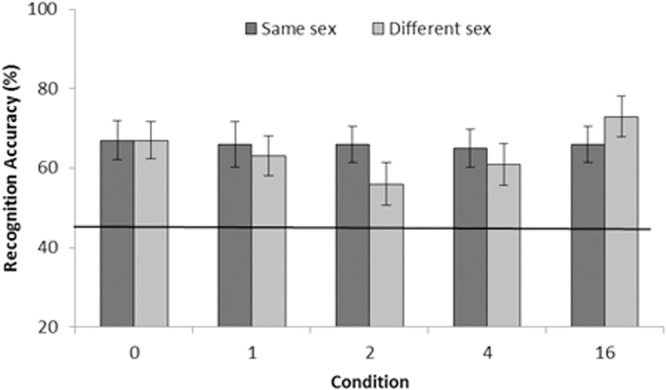
Test accuracy as percentage correct (with SEM) from Experiment 3. Data are organised by number of different comparators presented (0, 1, 2, 4, or 16), and are presented as a function of exposure condition (same sex and different sex). The solid line represents the baseline brief exposure control from Experiment 3B. Accuracy for the brief exposure was 45% (SEM 3.7).

Fig. 4 displays the percentage of correct responses as a function of exposure condition (same sex and different sex) and the number of different comparator presented (0, 1, 2, 4, & 16). Inspection of the figure suggests that performance was equivalent across conditions and number of comparators. A within-subjects ANOVA with the factors of condition type (same sex and different sex) and number of comparators confirmed that there were no significant effects on accuracy due to exposure condition, *F*(1, 39) = 0.42, *p* = .521, MSE = 0.073, *B_01_* = 6.614; or to the number of comparators *F* (4, 156) = 1.00, *p* = .409, MSE = 0.096. No significant interaction between condition type and number of comparators was found *F*(4, 156) = 0.80, *p* = .525, MSE = 0.100. To the extent that adding comparators should increase accuracy, the simple effects analysis comparing the performance of each number of comparator exposures are reported. Beginning with the control condition (0 comparators), no differences were found between: 0 and 1 (*F*(1, 39) = 0.22, *p* = .639, MSE = 0.112, *B_01_* = 7.275), 0 and 2 (*F*(1, 39) = 1.59, *p* = .215, MSE = 0.099, *B_01_* = 3.791), 0 and 4 (*F*(1, 39) = 0.79, *p* = .377, MSE = 0.096, *B_01_* = 5.508), or 0 and 16 comparators (*F*(1, 39) = 0.34, *p* = .562, MSE = 0.073, *B_01_* = 6.686). In addition no differences were found between the maximum number of comparators (16) and the following amount of comparators: 4 (*F*(1, 39) = 2.56, *p* = .117, MSE = 0.074, *B_01_* = 2.402), 2 (*F*(1, 39) = 4.06, *p* = .051, MSE = 0.075, *B_01_* = 1.226), or 1 (*F*(1, 39) = 1.11, *p* = .299, MSE = 0.090, *B_01_* = 4.755). No other differences between any other number of comparator exposures were observed: 1 vs. 2 (*F*(1, 39) = 0.43, *p* = .514, MSE = .130, *B_01_* = 6.572), 1 vs. 4 (*F*(1, 39) = 0.11, *p* = .740, MSE = 0.252, *B_01_* = 7.684), 2 vs. 4 (*F*(1, 39) = 0.16 *p* = .691, MSE = 0.088 *B_01_* = 7.505).

Non-parametric analysis these results confirmed the conclusions of ANOVA analysis. That is, there was no significant difference in accuracy number of comparators *χ^2^*(4) = 4.03, *p* = .403 or exposure condition *χ^2^*(1) = 0.68, *p* = .414. Post-hoc analysis revealed that there was no differences between number of comparators (smallest *Z* = 1.88, *p* = .060 between 2 and 16 comparator exposures). This analysis again closely reflected that of the parametric analysis.

It should be noted that although the difference between the 16 comparators and 2 comparator condition, reported in the parametric analysis, is approaching standard levels of significance (*i.e., p* = .051) the Bayesian analysis provides inconclusive evidence to support either the null or alternative hypothesis. Moreover, this result is driven by poor performance in the dissimilar comparator condition and therefore does not speak to the theoretically relevant effects of comparison between similar stimuli.

## Experiment 3B

Experiment 3B used the same 20 target faces and similar associated test procedures as in Experiment 3A. However, participants only received one brief (2 s) exposure to the target stimuli prior to test (for half of the stimuli it was followed by a same sex comparator and for the other half by a different sex comparator). This corresponds to the brief exposure condition from Experiment 2 and so provides a baseline to which performance in Experiment 3A can be compared.

### Methods

**Participants, apparatus & stimuli**. A total of 12 participants, 10 females and 2 males (mean age 20.1, range 18–24) were recruited from the School of Psychology at Cardiff University. All were undergraduates who participated in return for course credit. None of the 12 had participated in any of the previous experiments described here. All apparatus and stimuli were identical to those used in Experiment 3A.

**Design and procedure**. There were two conditions of exposure, both displayed a target and comparator for 2 s before the test arrays were presented: Brief same sex, in which a target was followed by same sex comparator, and brief different sex, in which a target was followed by a different sex comparator. There were eight targets, 4 male and 4 female, with two target faces from each sex per condition. Blocks of testing were counterbalanced such that each condition was presented first and second equally often. Similarly each sex was presented first and second equally often. All other details, including definitions of same sex and different sex, were identical to those of Experiment 3A.

### Results

A preliminary analysis from Experiment 3B confirmed that, after brief exposure, there were no significant differences in accuracy between the stimuli exposed with either a single same sex or a different sex comparator, *t*(11) = 1.65, *p* = .125, *B_01_* = 1.461.

Comparisons between brief and repeated exposure can been seen in Fig. 4. The dotted line represents performance across conditions from Experiment 3B. A between-subject *t*-test revealed that performance collapsed across all conditions in Experiment 3A was superior to performance across conditions in Experiment 3B, *t*(50) = 5.22, *p<*.001, *B_01_*< 0.001. This difference was also observed in a separate analysis comparing each condition from Experiment 3A to the baseline of brief exposure from Experiment 3B. That is, repeated exposure to same sex stimuli produced better performance than brief exposure, *t*(50) = 4.51, *p<*.001, *B_01_*< 0.001, as did exposure to different sex stimuli, *t*(50) = 4.62, *p<*.001, *B_01_*< 0.001. It is also worth noting that further analysis examined separate performance for each number of comparators to the baseline performance of 3B. Although not reported fully here, the results demonstrate, as anticipated, that all repeated exposure conditions produced better performance than the baseline (smallest *t*(33) = 3.07, *p* = .004, *B_01_* = 0.132, between 2 comparators (3A) and the baseline brief control (3B)).

### Discussion

The results of Experiments 3A and 3B demonstrate that repeated exposure produced an advantage over brief exposure, and that performance with targets exposed with same sex comparators was equivalent to those with different sex comparators. Moreover, the accuracy of target selection during test was not influenced by altering the number of comparators during exposure. In other words, performance following exposure with the maximum number of comparators (*e.g.*, sixteen) showed no advantage over targets without any comparators. Again these interpretations were strengthened by the Bayesian analysis for the critical comparisons. Therefore, taken together, Experiments 3A and 3B replicated the key findings of Experiment 1 and 2. Equivalent learning was observed across repeated exposures regardless of comparator type.

## General Discussion

The three experiments reported here examined the potential beneficial effects of similarity and the opportunity for comparison [25–28] on the ability to select a familiarised individual from an array of novel faces. In Experiments 1 and 2, exposure to a target face along with comparators of the same sex (either morphed to be explicitly similar to the target or not) produced no facilitation in selecting the target, relative to target faces that were exposed with either different sex comparators or no comparators at all. In Experiment 3, increasing the number of comparators during the exposure phase also produced no improvement in test performance. Importantly, the failure of comparison to improve identification accuracy cannot be attributed to a general failure of learning as performance in both similar/same sex comparator conditions, and repeatedly exposed controls, was superior to brief exposure conditions. It is important to note that these null results are informative with respect to the absence of a practically useful comparison effect facilitating learning to identify a target face. Firstly, these null results were obtained despite the fact that all of the experiments were well-powered to detect the sort of large effects that would be most useful in a practical setting, and would have been predicted by perceptual learning analyses of comparison. Secondly, the Bayesian analysis of each experiment suggested that the observed results were in favour of no underlying effect existing compared to alternative models where there was an underlying effect. In short, the fact that there was no facilitation, despite the differing levels of similarity and the varying numbers of comparators used during exposure, suggests that there is no practical beneficial effect of comparison on the processing of unfamiliar faces above that of repeating exposure to a face.

Before considering the implications of these results in detail, the concern was raised during review that the naming of the target stimuli during the exposure phase could have compromised the effects of comparison. In particular, it was suggested that presenting a target face along with similar comparators might do two things: (i) improve the processing of that face image, and (ii) increase generalisation between the target and its comparator images (possibly because the presentation of the images close together might lead to the formation of associations between the name of the target and the non-target comparators, and between the target and the label “not-target”). These two effects could cancel each other out and thus produce no net benefit for comparison over simple presentation of the target alone. This concern appears to us to be misplaced. Firstly, our key concern here is practical—whether comparison helps us learn to recognise an individual from their photo. Because this is explicitly an issue about identification, then it makes pragmatic sense to focus the experimental tasks on identity. The real importance of the current experiments lies in the fact that they offer no support whatsoever for the idea that comparison could be a practical means of learning to selectively identify a particular individual. Secondly, the removal of the name identification would not necessarily impact on processes (i) and (ii)—for example, there is no need to know that one stimulus is the target in order to form associations between the face images that are exposed close together in time. Indeed, the literature on sensory preconditioning (*e.g.* [45, 46]) shows that the pairing of two stimuli is sufficient to support generalisation of what is learnt about one of them to the other. Thus the possibility that alternating a target image with similar comparators could engage processes that support and oppose discrimination is inherent in the intermixing of the stimuli, not the naming of the target. Regardless of the exact mechanisms involved, the current results provide no support for the idea that providing the opportunity to compare a target image to similar face images might facilitate the subsequent selection of that target from novel foil faces.

The idea that comparison-based exposure effects do not enhance matching when testing against novel foils (as demonstrated here) does not mean that this process cannot contribute to discriminating between a set of faces. The work of Mundy *et al*. [1] demonstrates that comparison, during exposure training, does help facilitate discrimination performance when the faces to be discriminated at test were the same as those presented during the exposure phase (see also [19, 25, 47, 48]). Similar effects have also been demonstrated using identical twins [49], in which case the enhancement in discrimination transferred to new images of the same twins (see also [50]). In this light, the current experiments suggest that the facilitatory effects of comparison will be strongest when discriminating between a target and exposed comparators, and may even not extend to novel test foils at all. This is consistent with the findings of Blair and Hall [29] whereby rats were exposed to two compound flavours AX and BX on an intermixed schedule (AX, BX, AX, BX, …) before a taste aversion was established to AX. This aversion did not generalise to BX at test demonstrating good discrimination between the exposed stimuli. However, the aversion did generalise to a novel test stimulus (CX), suggesting no improvement in discrimination performance between an exposed target and a similar stimulus that were not exposed in alteration. Thus, it seems that comparison may aid certain discriminations, but this does not easily extend to better identification against non-exposed foils.

Against this background, and the current results, the fact that Dwyer and Vladeanu [34] found that matching of a target against novel test foils was improved by the presence of similar comparators requires explanation. The simplest possible explanation is that the results of Dwyer and Vladeanu [34] may be a statistical false positive. While this cannot be ruled out, it should be remembered that a large number of perceptual learning studies that demonstrate comparison effects through the advantage for intermixed over blocked exposure schedules (*e.g.* [1, 19, 21, 25, 48]). So the possibility that comparison effects *per se* are false positives seems unlikely. Thus the more relevant question is whether the divergence of results might be attributable to any of the differences between the stimuli used in the current experiments and those of Dwyer and Vladeanu [34]. As noted in the introduction, the stimulus set used by Dwyer and Vladeanu consisted of artificially generated faces whereas our stimuli consisted of face photographs. These generated stimuli may have belonged to a distinctive or restricted portion of face-space. In this light, the difference between these sets of results could be attributed to the way in which the stimulus set of Dwyer and Vladeanu were generated using a computer program which models each face upon an average. That is, each face produced from the modelling software is framed upon an average model of a collection of input faces used to build the model (see, [51–53] for a detailed explanation of how some of these models are constructed and a discussion of how they work). It follows from this that any face produced by the model will be defined using a constrained amount of dimensions. Essentially, this means that all stimuli created using this method would belong to a theoretically smaller ‘space’ than real faces. If we again consider the idea of the face-space framework, the dimensions which span this space are assumed to encode physical or abstract attributes that render different faces discriminable from one another [54]. Moreover, if we consider estimates of the amount of dimensions as between 15–22 [36], then reducing these dimensions may have facilitated a viewing strategy whereby exposure highlighted the crucial differences on a small number of dimensions allowed for easier identification in the matching task. That is, matching becomes easier because the modeller, constrained by the collection of input faces, creates all stimuli from this reduced space. In turn, the similar condition allowed a more focused approach on the perceptually relevant information with which to identify an individual. Conversely our experiments, using real face images wherein the dimensions are much less restricted, failed to produce any similarity advantage because there is no way of ensuring that information critical to distinguishing the target and similar comparators would be equally informative when distinguishing the target from novel test foils. That is, the inherent complexity of real face stimuli might well mean that considering them as simply a collection of common and unique elements (as is common in the perceptual learning literature) may not be a useful heuristic considering the multitude of ways faces can differ or be similar. In terms of a face-space model [35, 55], the differences between faces are represented by how they differ on all the dimensions within this space. That is, the comparisons during exposure may have covered certain dimensions, but these dimensions may not be informative with respect to discriminating the target from other faces. Another way to phrase this situation is that while comparison may well help identify how ‘Tom’ differs from ‘Bob’ it is unlikely to be informative for how ‘Tom’ differs from ‘Fred’. Returning to the Iraqi playing cards, comparison between them may well help identify how Deputy Prime Minister Tariq Aziz differed from defence minister Ali Hassan al-Majid, it is unlikely to be informative for how Tariq Aziz differed from a randomly selected person on the streets of Baghdad. Therefore, the above analysis suggests that comparison to a limited number of comparators is unlikely to identify the critical aspects of a face on enough relevant dimensions. With this in mind, the current experiments may have been unable to capture enough similarity between a target and comparators to be informative when novel foils were introduced. As such, it is certainly possible that greatly increasing the number of comparator faces used in the exposure phase may provide enough information to facilitate subsequent target identification to some degree. However, as the number of comparison faces increases, the practical tractability of the comparison process decreases.

The findings presented here can be considered in the forensic or applied settings outlined earlier. Proving our identity with some form of photographic identification is a vital aspect of our daily routine, despite the fact that there is clear evidence that people are ineffective and error-prone at this task [3, 4]. It is of particular importance that ways to overcome this deficiency are explored. The current results support previous suggestions [18] that repeated brief exposure to an unfamiliar face will support at least some ability to reliably identify an individual from their photograph (also see [6] for an examination of how feedback can improve unfamiliar face matching). Unfortunately, despite evidence of strong perceptual learning based improvements in a wide range of ‘real-life’ situations such as viewing X-ray images [56], wine tasting [57] and chick sexing [58], our current results suggest any direct forensic application of comparison-based perceptual learning for face recognition is limited. That is, despite the plausible implications from perceptual learning theory and the suggestive results from artificial face stimuli, the opportunity for comparison between a target face and other images does not improve the learning of new faces in a practically tractable fashion through a process of perceptual learning.

## Supporting Information

S1 Data JDL Raw data for each participant in three experiments. Data includes Accuracy and Confidence scores for each condition.
(XLS)
